# The Influence of Changes in Lifestyle and Mercury Exposure in Riverine Populations of the Madeira River (Amazon Basin) near a Hydroelectric Project

**DOI:** 10.3390/ijerph110302437

**Published:** 2014-02-26

**Authors:** Sandra S. Hacon, José G. Dórea, Márlon de F. Fonseca, Beatriz A. Oliveira, Dennys S. Mourão, Claudia M. V. Ruiz, Rodrigo A. Gonçalves, Carolina F. Mariani, Wanderley R. Bastos

**Affiliations:** 1 Escola Nacional de Saúde Público Sergio Arouca—Fundação Oswaldo Cruz, Rua Leopoldo Bulhões, 1480 Manguinhos, Rio de Janeiro, 21041-210, Brazil; E-Mails: beatrizenf@gmail.com (B.A.O.); dennys@ensp.fiocruz.br (D.S.M.); 2 Faculdade de Ciências da Saúde—Universidade de Brasília, C.P. 04322, Campus—Asa Norte, Brasília, Distrito Federal, 70919-970, Brazil; E-Mail: jg.dorea@gmail.com; 3 Instituto Fernandes Figueira—Fundação Oswaldo Cruz, Av. Rui Barbosa, 716 Flamengo, Rio de Janeiro, 22250-020, Brazil; E-Mail: marlon@iff.fiocruz.br; 4 Laboratório de Química da Pontifícia, Universidade Católica do Rio de Janeiro, R. Marquês de São Vicente, 225. Gávea, Rio de Janeiro, 22451-900, Brazil; E-Mails: claudveg@yahoo.com (C.M.V.R.); rodrigoagoncalves@gmail.com (R.A.G.); 5 Santo Antônio Energia, Rua Tabajara, 824—Olaria, Porto Velho, Rondônia, 76801-316, Brazil; E-Mail: carolinamariani@santoantonioenergia.com.br; 6 Laboratório de Biogeoquímica Ambiental Wolfgang C. Pfeiffer, Universidade Federal de Rondônia, Porto Velho, RO, 76801-974, Brazil; E-Mail: wanderbastos@yahoo.com.br

**Keywords:** mercury in hair, fish intake, riverine communities, Amazon basin

## Abstract

In the Amazon Basin, naturally occurring methylmercury bioaccumulates in fish, which is a key source of protein consumed by riverine populations. The hydroelectric power-plant project at Santo Antônio Falls allows us to compare the Hg exposure of riverine populations sparsely distributed on both sides of the Madeira river before the area is to be flooded. From 2009 to 2011, we concluded a population survey of the area (N = 2,008; representing circa 80% of community residents) that estimated fish consumption and mercury exposure of riverine populations with different degrees of lifestyle related to fish consumption. Fish samples from the Madeira river (N = 1,615) and 110 species were analyzed for Hg. Hair-Hg was significantly lower (*p* < 0.001) in less isolated communities near to the capital of Porto Velho (median 2.32 ppm) than in subsistence communities in the Cuniã Lake, 180 km from Porto Velho city (median 6.3 ppm). Fish Hg concentrations ranged from 0.01 to 6.06 µg/g, depending on fish size and feeding behavior. Currently available fish in the Madeira river show a wide variability in Hg concentrations. Despite cultural similarities, riparians showed hair-Hg distribution patterns that reflect changes in fish-eating habits driven by subsistence characteristics.

## 1. Introduction

Methylmercury (MeHg) is a known neurotoxicant that accumulates in the food chain posing a significant risk to fish consumers, mainly women of childbearing age and young children. In the Amazon, naturally occurring MeHg bioaccumulates in fish, which is a key source of protein and essential nutrients in a cassava-based diet [1]. The habitual high fish intake of traditional riverine communities in the Amazon Basin represents a unique scenario for studying human exposure to mercury (Hg), its interaction with endemic diseases, and the metabolism of toxic substances [2].

Fish consumption is high across all Amazonian populations, resulting in a relative increase in hair-Hg concentrations [3]. Dependence on fish consumption has showed to be a function of the traditional lifestyle associated with the isolation of Amazonian communities. Increase in hair-Hg concentrations as a function of distance to urban center centers was observed for subsistence villagers of Rio Negro [4]. Indeed, in the neighboring Bolivian Amazon, fish consumption tends to be higher among the more isolated and impoverished inhabitants [5].

The traditional lifestyle of riverines living in communities along the Madeira river Basin has been impacted by economic development in the last 40 years [6]. First came the gold rush of the 1980s, and then came the opening of roads, deforestation for agricultural projects, and construction of a great number of hydroelectric dams resulting in displacement and changes in traditional subsistence lifestyles. For all this region the Brazilian government has planned approximately 80 hydroelectric plants. Regardless of the scheduled date of construction, this activity will flood approximately 3% of the Amazon forest [6,7]. These programs and projects, along with immigration spurred by economic activities, are bringing rapid urbanization and changes in lifestyle, gradually replacing the traditional food supply chain that depended heavily on fish and natural foods abundant in the forest [8]. 

The rural inhabitants of the Madeira river, in Brazilian territory, have shown more dependence on fish, and as a result have consistently high hair-Hg concentrations [3]. The hair-Hg (HHg) concentrations of riverines living in the Madeira River Basin have been reviewed by Barbieri and Gardon [3]. In Bolivian territory, mean hair-Hg ranged from 1.9 to 8.7 ppm, whereas in Brazilian territory mean hair-Hg ranged from 10.2 to 15.2 ppm [3]. The high HHg values among Brazilian riverines represent a *per capita* fish consumption of 7 meals/week, contrasting with urban mothers with low fish consumption (1 meal/week) and attendant mean hair-Hg (5.4 ppm) concentration [3].

The concentrations of mercury in fish of the Amazon Basin vary greatly. A systematic comparison is riddled with difficulties related to fish habitat diversity, incomplete information (differences in local fish names from region to region, and insufficient knowledge of the fish feeding hierarchy) that includes fish size (length or weight) and age, which are important in controlling the random nature of fish sampling [9]. Indeed, when comparing rivers of the Amazon Basin, there are no salient features distinguishing fish from rivers impacted by intense gold-mining activities from the past, like the Madeira river, from fish caught in non-impacted waters [9]. Traditional subsistence villagers of the Madeira river are among the largest group of Amazonians in *per capita* fish consumption (*ca.* 148.2 kg/year); because of this elevated consumption, seasonal variability in fish availability has little impact on overall hair-Hg concentrations throughout the year [10].

The aim of this study is to assess mercury exposure, fish mercury concentrations and fish consumption habits in riverine populations inhabiting both sides of the Madeira river near the Santo Antônio hydroelectric power-plant, before flooding of the reservoir. 

## 2. Experimental Section

### 2.1. Background

This cross-sectional descriptive study is part of a large interdisciplinary research project to integrate social, environmental, and health assessment of all riverine inhabitants of an area likely to be impacted by the Santo Antônio hydroelectric power-plant. This hydroelectric power-plant is being constructed at the Santo Antônio Falls, which is the last waterfall before Porto Velho city (state capital of Rondonia) and marks the beginning of the navigable stretch of the Madeira river until the Atlantic Ocean. The current National Developmental Program of the Brazilian Federal Government has planned the construction of two hydroelectric power-plants along the Madeira river in the state of Rondônia (Western Amazon): Santo Antônio (about 7 km upstream from Porto Velho city) and Jirau (120 km upstream from Santo Antônio falls). The Madeira river is the second largest river in the Amazon River Basin, and is its main tributary. It runs 1,459 km in Brazilian territory, has an average flow of 31,200 m^3^ per second (5,000 to 45,000 m^3^·s^−1^) and transports one of the largest loads of sediments in the world [11]. The research protocol, survey questionnaires and procedures were reviewed and approved by a Brazilian Research Ethics Committee (CAAE: 0010.0.047.000-09). After a comprehensive explanation, including the assurance that their involvement in this study was voluntary, consent forms were signed by the participants.

### 2.2. Area of Study and Population

Data were collected from May 2009 to April 2011 in the pre-impoundment phase of the Santo Antônio reservoir. Adults and children from riparian communities upstream and downstream of the dam project were surveyed. Fish were sampled in the area of Madeira river representing the most available species, which reflects the most consumed by the riverines. 

**Figure 1:**
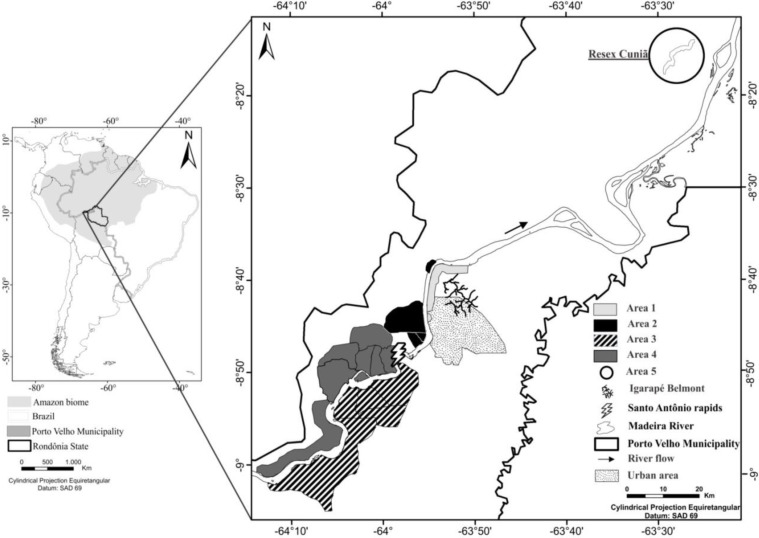
Illustration of the study area showing riparian communities regarding their proximity and location around the Santo Antônio falls. Area 1 (downstream the rapids, right bank), Area 2 (downstream the rapids, left bank), Area 3 (upstream the rapids, right bank), Area 4 (upstream the rapids, left bank) and the extractive reserve of the Cuniã Lake (Area 5), about 180 km downstream the rapids. Porto Velho city is the capital of Rondônia State.

Figure 1 shows the study area, illustrating the four grouping of riparian communities regarding their proximity and location around the Santo Antônio falls where the dam project was being constructed. The studied communities occupy an area extending from Porto Velho city (8º47'31''S and 63º57'7''W) to 220 km in both directions (up- and downstream) from the dam construction site. Some communities are more isolated or have fewer resources than others (*i.e*., energy, transportation, access to health services and education, and distance to food trade centers or grocery stores). We invited the all families from the communities upstream and downstream with direct influence from the projected dam to participate in the study. About 630 families that were invited and 495 families agreed to participate on a voluntary basis. The inclusion criterion was to have lived in these communities for at least one year. The exclusion criteria were any evident major neurological disease (auto referred, guaranteed or diagnosed by one of the researcher physicians) or those who refused to participate at any moment.

We grouped the communities into five areas, taking into account the left and right banks of the river, as well as spatial considerations regarding upstream and downstream. All the communities in the left bank had more difficulties to access transport, schools, health services and food variety than those from the right bank. Area 1 is located on the right bank downstream from the falls and incorporates, mainly, Belmont village (closest to the city of Porto Velho) with access by roads to the capital city on the right bank of Madeira river. Area 2 (left bank, downstream from the falls) comprises four villages close to the city of Porto Velho by boat (Novo Engenho Velho, São Sebastião, Balsa and Niterói villages). Area 2 has more difficulties to access roads and industrialized food. Area 3 (upstream from the falls on the right bank) comprises the five villages of Teotônio, Maria Auxiliadora, Princesa, Paulo Leal and Morrinhos with similar traditional food habits based in fish consumption, but more isolated than Area 2, having difficult access the main highway and to services and goods available in the capital city of Porto Velho. Area 4 (upstream from the falls on the left bank) comprises seven villages (Morrinhos, Amazonas, São Domingos, Cachoeira dos Macacos, Renascer, Ilha do Jatuarana, and Porto Seguro) that are more isolated than other villages. As a reference for typical traditional riverine living, we included an isolated community located in Cuniã Lake on the left side of the Madeira river (Area 5). This community is in a remote area, 180 km downstream from the capital city of Porto Velho. The ecological complex of Cuniã consists of more than 60 interconnected lakes in a total area of 55,850 ha and has a total estimated population of 309 individuals. It is the most remotely located of all assessed villages, with no means of regular transportation in the middle of the forest. Altogether, 2,008 individuals answered questionnaires and 1,945 individuals had hair samples collected in the studied areas. 

### 2.3. Questionnaire and Fish Intake Assessment

A semi-structured questionnaire was applied to adults by trained interviewers; in all households with children an adult was invited to participate of the interview to collect information. Information concerning socioeconomic status, life style, demographics, occupational history, refereed morbidity and a dietary survey (including species of fish frequently consumed, and the consumption on the last 24 h) was assessed. A specific question was about how many times the interviewed did eat fish last week. In general, the questionnaires took approximately 1 h. The common names of fish species referred to by the interviewees were then classified according to the study by Bastos *et al*. (2008) [12] and, then, categorized with respect to mean total-Hg concentrations.

### 2.4. Total-Hg Determination in Hair

At the time of the interview we also collected a sample of hair cut from the occipital area near to the scalp. Total Hg concentration (hair-Hg) was determined in individual hair samples according to routine laboratory procedures at the Wolfgang Christian Pfeiffer Environmental Biogeochemical Laboratory (BIOGEOQ) at the Federal University of Rondônia (UNIR). Briefly, after mineralization in acid-oxidant medium, total Hg determination was performed by cold vapor atomic absorption spectrometry on a Perkin-Elmer (Ueberlingen, Germany) FIMS-400**^®^** instrument [13]. For quality control, all analytical runs included material certified by the International Atomic Energy Agency (IAEA-085 and IAEA-086). Recovery rates were above 80% and detection limit below 0.03 mg·kg^−1^. 

### 2.5. Fish Sampling and Total-Hg Determination

Fish samples were collected throughout the Madeira River Basin, covering a stretch of 230 km (downstream and upstream of the falls) and from Cuniã Lake. Fish specimens (N = 1,599) were captured at the time of the study and taxa were identified by the Ichthyofauna Laboratory of the University of Rondônia according to Santos *et al*. [14].

Determination of total Hg concentration in fish muscle was done according to routine procedures developed by Bastos *et al*. [13]. Briefly, fish samples were mineralized in an acid-oxidative medium and digested for 60 min in a block digester at 80 °C (Tecnal, Mod. 007A, São Paulo, Brazil). Total Hg measurements were carried out by cold vapor atomic absorption spectrophotometry (Flow Injection Mercury System, Perkin Elmer FIMS-400**^®^**). Precision and accuracy were ensured by using internal standards against certified reference materials (Dogfish Muscle, DORM-2, National Research Council of Canada, Ottawa, ON, Canada), which have been used in intercalibration exercises among Brazilian laboratories showing detection limit below 0.001 mg kg^−1^. Daily calibration and method verification was done with known concentration standards. 

### 2.6. Data Analysis

The characteristics of the communities, the fish consumption habits and the HHg concentration and Hg concentration in different fish species were analyzed using descriptive statistics. The results of HHg were stratified by age groups and the hair Hg values were expressed as median and the 5 and 95th percentiles. Because of non-normal data distribution of main variables, data analysis was based on non-parametric statistical tests. The Spearman´s correlation analysis was applied to assess association between fish consumption and HHg. Graphics and statistics were performed using SPSS^®^ 20.0 software for MAC. Statistical results were considered significant when *p* < 0.05.

## 3. Results and Discussion

The sample was composed by 2,008 individuals who answered questionnaires and 1,945 individuals had hair samples collected in the studied areas. From the 630 families living in the selected areas, 495 families agreed to participate with a total of 1,945 individuals. The general acceptance rate of the communities ranged from 79% to 90%. The sample population consisted of 41% riverside children and teenage and 59% of adults, being 41% of female and 51% of male. 

### 3.1. Characteristic of the Riverine Population and Exposure

In general, some socio-demographic and socio-economic characteristics of the riverine communities were similar. Spatially, all the communities live on the river banks. However, their access to the urban area may influence on their fish consumption. This was the reason why we considered separately the left and right banks of the Madeira river in this study. For the communities living in the left bank, it is more difficult to access other sources of protein besides fish consumption. Table 1 summarizes general characteristics of the population. 

**Table 1:** Characteristics of the study population.

Grouped Villages	Area 1	Area 2	Area 3	Area 4	Resex Cuniã
**Position along the Madeira river**	Downstream the waterfalls	Downstream the waterfalls	Upstream the Santo Antônio waterfall	Upstream the Santo Antônio waterfall	Downstream the Santo Antônio waterfall
**Riverside margin**	Right	Left	Right	Left	Lakes on left
**Access to urban area in Porto Velho city**	7–10 km away from the urban area; road available in dry seasons; viable by bike	8–15 km away from the urban area; road available in dry seasons; viable by bike (ferry available daily)	20–30 km away from the urban area; road available	25–50 km from the urban area; road available in dry seasons until the ferry in Area 2	180 km from urban area; no road available; viable only by boat (very difficult in dry season)
**Main source of fish**	Belmont **	Madeira river	Madeira river	Madeira river	Cuniã lake
**Assessed individuals (total; % of children)** *	276; 40.6%	343; 46.4%	667; 43.2%	471; 39.7%	251; 45.8%
**Residence time on site (years)**	16 (1–50)	21 (1–47)	15 (2–53)	14 (2–52)	30 (4–63)
**Sewer system and safe drinking water supply**	No	No	No	No	No
**Local primary-health-care unit**	No	Yes	Yes	No	No
**Education of the adults in years (proportional% frequency)**					
**Illiterate**	11.0%	8.5%	9.7%	11.8%	7.8%
**1** **–** **3 years (literate)**	10.4%	17.0%	21.2%	20.9%	13.5%
**4–9 years**	49.2%	51.2%	56.9%	55.2%	65.3%
**10 years or more**	29.4%	23.1%	12.3%	11.1%	13.5%
Family income (R$/month) ***	1000 (500–2,000)	1000 (460–2,750)	850 (300–5,000)	930 (300–6,000)	600 (200–3,500)
**Main occupation of adults (proportional % frequency)**					
**Fishing**	6.1%	11.1%	19.0%	27.9%	29.3%
**Goldmining**	6.1%	0%	0.3%	0%	0%
**Farming**	11.0%	6.0%	21.8%	18.6%	11.4%
**Extractivism**	0%	0%	0%	0.8%	0.7%
**Building**	2.5%	1.5%	0.9%	0%	0%
**Studying **	6.1%	6.0%	6.0%	4.6%	6.4%
**Home**	25.8%	30.7%	22.2%	27.5%	20.0%
**Variable (seasonal)**	47.9%	44.7%	29.7%	20.5%	32.1%

Notes: Santo Antônio waterfall (rapids) is the last before Porto Velho city and marks the beginning of the navigable stretch of Madeira river; Values expressed as median (5–95th percentiles); * Adults when age > 16 year; Children when age < 17 year; ** Belmont stream (Belmont igarapé) is the main tributary of the Madeira river in the area immediately downriver from the Santo Antônio waterfall; *** R$ = BRL = Brazilian Real; Family income = ∑ income within family.

The areas with the most difficult access or most distant from the city of Porto Velho showed a proportionally higher number of adults engaged in fishing activities (Area 5 > Area 4 > Area3), and proportionally more people with <10 years of schooling, and lower family income; they tended to depend more on the river and the forest for their livelihood and are the ones with higher median hair-Hg concentrations. Overall, these areas shared a simple life, housing without basics indoor sanitary facilities, lack of access to safe drinking water supply; in only two (Areas 2 and 3) there were limited primary health-care facilities. In all studied areas the residence time ranged from 1 to 63 years. The Areas 4 and 5 are the most isolated ones. 

**Figure 2:**
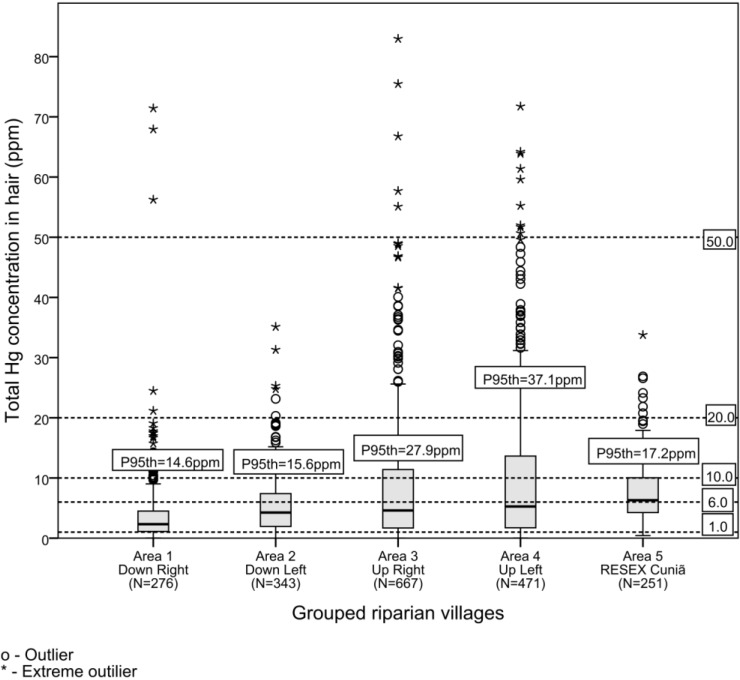
Box-plot representation of total hair-Hg concentrations in the studied population (ppm w/w = mg·kg^−1^).

A summary of data on frequency of fish consumption and attendant Hg exposure (HHg) is shown in Table 2 and Figure 2. The study showed that in these riverine communities daily fish consumption is more prevalent than in the other areas. To better illustrate the overall HHg of each area we organized the data into: preschool children (0 to 5 years), children (6 to 15 years), adults of both sexes (>16 years); we also categorized women of childbearing age because of vulnerability to prenatal exposure to fish-mercury (16 to 49 years). 

**Table 2:** Regular fish consumption and total-Hg concentration in riparians’ hair from Madeira River Basin.

Grouped Villages	Area 1	Area 2	Area 3	Area 4	Resex Cuniã
**Fish consumption**					
Never eat fish	5.1%	3.0%	2.9%	2.4%	0.0%
Fish 1/15 days	27.0%	20.1%	20.6%	17.1%	1.6%
Fish 3–5/week	43.1%	36.4%	32.1%	33.2%	19.4%
Fish > 3/week	19.0%	27.2%	18.2%	24.2%	21.4%
Daily eat fish	5.8%	13.3%	26.1%	23.1%	57.7%
**Hair-Hg (ppm)**					
Age ≤ 5 years	1.9 (1.4–2.4)/3 *	2.01 (0.4–8.4)/10 *	3.7 (0.4–23.2)/52	4.2 (0.4–43.6)/42	4.9 (0.9–14.4)/30
Age 6–16 years	1.9 (0.2–8.2)/109	3.9 (0.4–14.7)/136	3.6 (0.4–31.7)/225	4.1 (0.2–35.3)/135	5.3 (1.7–14.3)/84
Age > 16 years	2.9 (0.2–17.7)/162	4.8 (0.4–16.7)/182	5.3 (0.3–27.6)/364	5.7 (0.5–37.2)/276	8.2 (1.7–19.4)/135
Female 13–49 years **	2.4 (0.3–17.5)/156	3.5 (0.4–10.8)/164	4.0 (0.3–26.1)/307	3.5 (0.2–26.8)/204	5.3 (1.6–13.4)/111
Altogether	2.3 (0.2–14.6)/274	4.3 (0.4–15.6)/328	4.6 (0.3–27.9)/641	5.3 (0.3–37.1)/453	6.3 (1.7–17.2)/249
Comparing areas ***	1	2	2–3	3–4	5
Rank correlation ****	0.239 (*p* < 0.001)	0.105 (*p* = 0.059)	0.056 (*p* = 0.158)	0.113 (*p* = 0.018)	0.120 (*p* = 0.061)

Notes: Age limits included. Hair-Hg values expressed as median (5–96th percentiles)/number of individuals whose hair sample was analyzed; * Range exceptionally expressed as minimum–maximum; ** Usual female’s childbearing age considering mean menopause age = 50 years [15]; *** The numbers (1–5) represent significantly different groups at Mann Whitney U test with respect to hair-Hg concentration; **** Spearman’s rank correlation analysis between fish consumption and hair-Hg concentration: rho correlation coefficient (*p* value).

Reported daily fish consumption was substantially higher for the Cuniã riverines (*ca*. 58%), relatively less for Areas 3 and 4 (26% and 23% respectively), and much less for Areas 1 and 2. However, the pattern of HHg aggregated for each area showed that HHg concentrations were significantly higher in some than in others (Area 5 > Area 4 > Area 3). With respect to Hg exposure, the significantly lowest (HHg) was seen in Area 1 (*p* < 0.001) with both the lowest median and 95th percentile values (respectively: 2.3 and 14.6 ppm). Contrasting with the lower median HHg values found in Areas 1 and 2 (near Porto Velho city), Areas 3 and 4 showed the highest data dispersion, 95th percentiles. Considering all individuals (age groups and both sexes), fish consumption and HHg showed a statistically significant correlation only in Areas 1 and 4 with the correlation coefficient of 24% and 11%, respectively, as presented in Table 2.

Communities were grouped as upstream or downstream the dam and also as left or right riverside. Resex Cuniã: lacustrine reference area 180 km downstream Porto Velho. Boxplots: quartiles (Figure 2). One value (upstream the dam, left side) was excluded from the graphic because it showed atypical extreme value (124.3 ppm). Empirical limits: 1 ppm in hair of women of child-bearing age in the US, equivalent to the USEPA reference dose (RfD) of 0.1 µg/kg/day); 6 ppm (maternal hair-Hg associated to a 3-point decrement in IQ according [16,17]); 10 ppm (maternal hair-Hg associated with 5% risk for children with basis on Iraqi data); 20 ppm (twice the WHO limit for maternal hair) and 50 ppm (associated with a 5% risk of neurological damage to adults) [18].

**Figure 3:**
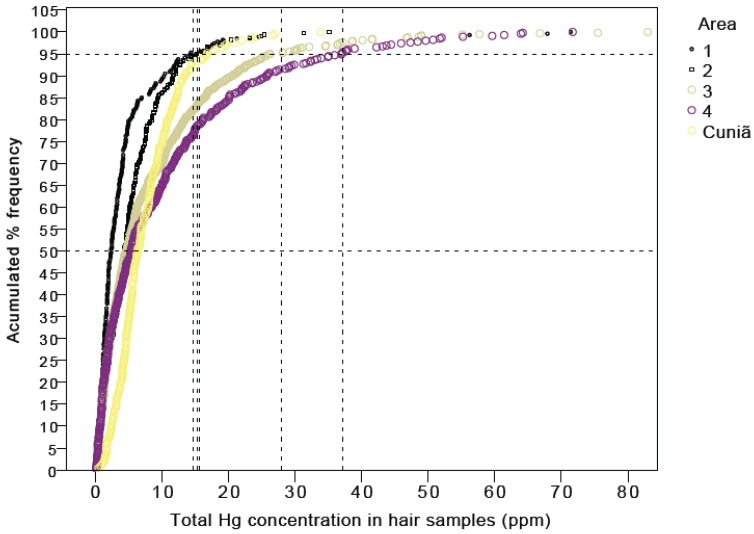
Percent distribution of hair-Hg concentrations illustrating the four village groups. Area 1 (downstream the rapids, right bank), Area 2 (downstream the rapids, left bank), Area 3 (upstream the rapids, right bank), Area 4 (upstream the rapids, left bank) and the extractive reserve of the Cuniã Lake (Area 5), about 180 km downstream the rapids. Porto Velho city is the capital of Rondônia State.

Figure 3 illustrates the distribution of HHg, reflecting the level of subsistence and/or traditionalism (fish consumption habits). Of the 1,945 analyzed hair samples, 17 individuals (0.9%) showed HHg > 50 ppm, 154 (7.9%) HHg > 20 ppm, 556 (28.6%) HHg > 10 ppm and 1,750 (90.0%) HHg > 1 ppm. Indeed, the most traditional riverines living in Cuniã (Area 5) showed the highest median HHg (6.30 ppm) compared to other riverine communities. In fact, in different age groups, the communities nearest the city of Porto Velho showed the lowest median HHg values. Regarding the pattern of distribution of HHg values, however, there were two extreme conditions shown in Figure 3: one is represented by communities in Areas 1 and 2, contrasting with the traditional Cuniã, showing a distinct pattern of HHg distribution; in this traditional community almost 70% showed HHg above 5 ppm. The other distant communities of Areas 3 and 4 showed respectively 55% and 50% of samples above 5 ppm.

### 3.2. Fish Mercury Concentration

**Figure 4:**
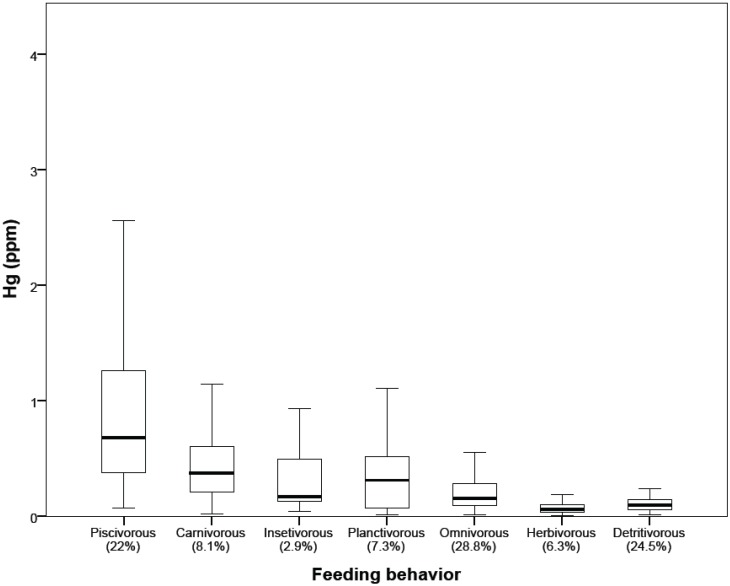
Box-plot representation of total-Hg concentrations in fish-muscle of 110 species caught at the 15 sampling sites as a function of fish feeding strategy.

Total-Hg concentrations in fish-muscle were species-specific; a detailed account of 110 species caught throughout the Madeira River Basin, covering a stretch of 230 km (downstream and upstream of the falls) and from Cuniã Lake is shown in Table 3. The Figure 4 summarizes abundance of catch and Hg concentrations as a function of fish feeding strategy. Species at the top of the food chain classified by groups (carnivorous and piscivorous) showed the highest median muscle Hg concentrations (respectively 0.68 ppm and 0.37 ppm), while species at the bottom of the food chain (detritivorous and herbivorous) showed the lowest median Hg concentrations (respectively 0.06 ppm, and 0.09 ppm). However, regarding abundance of fish (percent catch) the most frequent groups were not necessarily the ones with higher median Hg concentrations. Indeed, 53% of the catch was found in two groups (omnivorous, 28.8%; detritivorous, 24.5%) with the lowest median Hg concentrations (Figure 4).

**Table 3:** List of fish species (likely consumed) caught at 15 locations along the Madeira River Basin during the studied period and respective total Hg concentrations (µg/g).

Scientific Name	Feeding Behavior	N	Mean	Standard Deviation	[Hg], Median	Minimum	Maximum
*Acestrorhynchus sp.*	Carnivorous	77	0.41	0.28	0.37	0.09	1.27
*Ageneiosus sp.*	Carnivorous	21	0.41	0.29	0.39	0.02	0.88
*Anodus sp.*	Detritivorous	42	0.43	0.15	0.40	0.17	0.75
*Argonectes sp.*	Omnivorous	1	0.51		0.51		
*Auchenipterichthys sp.*	Omnivorous	120	0.11	0.06	0.10	0.04	0.54
*Auchenipterus sp.*	Insectivorous	35	0.39	0.26	0.35	0.10	0.93
*Brachyplatystoma sp.*	Carnivorous	114	1.58	1.13	1.33	0.12	4.89
*Brycon sp.*	Omnivorous	5	0.08	0.02	0.08	0.05	0.11
*Bryconops sp.*	Omnivorous	11	0.18	0.09	0.15	0.06	0.38
*Calophysus sp.*	Omnivorous	10	0.92	0.32	0.82	0.54	1.44
*Centromochlus sp.*	Insectivorous	2	0.22	0.09	0.22	0.16	0.29
*Chalceus sp.*	Insectivorous	2	0.11	0.01	0.11	0.11	0.12
*Cichla sp.*	Carnivorous	7	0.50	0.32	0.45	0.17	1.14
*Colossoma sp.*	Herbivorous	2	0.04	0.01	0.04	0.03	0.04
*Curimata sp.*	Detritivorous	15	0.09	0.04	0.07	0.04	0.16
*Curimatella sp.*	Detritivorous	11	0.07	0.03	0.06	0.04	0.11
*Cynodon sp.*	Carnivorous	7	0.89	0.44	1.08	0.26	1.47
*Geophagus sp.*	Omnivorous	4	0.18	0.06	0.21	0.09	0.23
*Hemiodus sp.*	Detritivorous	79	0.08	0.11	0.05	0.01	0.68
*Hemisorubim sp.*	Carnivorous	2	0.71	0.15	0.71	0.60	0.81
*Heros sp.*	Carnivorous	6	0.22	0.05	0.21	0.18	0.31
*Hoplias sp.*	Carnivorous	18	0.30	0.12	0.26	0.15	0.54
*Hydrolycus sp.*	Carnivorous	11	1.29	0.67	1.20	0.38	2.90
*Hypoclinemus sp.*	Insectivorous	3	0.25	0.11	0.31	0.13	0.32
*Hypophthalmus sp.*	Detritivorous	23	0.69	0.25	0.71	0.19	1.11
*Hypoptopoma sp.*	Detritivorous	14	0.04	0.01	0.03	0.02	0.05
*Hypostomus sp.*	Detritivorous	1	0.02		0.02		
*Ilisha sp.*	Carnivorous	2	0.88	0.01	0.88	0.87	0.89
*Jurengraulis sp.*	Detritivorous	22	0.09	0.05	0.07	0.05	0.26
*Laemolyta sp.*	Detritivorous	13	0.26	0.11	0.24	0.09	0.42
*Leporinus sp.*	Omnivorous	16	0.09	0.07	0.06	0.03	0.27
*Loricaria sp.*	Detritivorous	4	0.12	0.04	0.13	0.07	0.16
*Loricariichthys sp.*	Detritivorous	2	0.14	0.09	0.14	0.08	0.21
*Mesonauta sp.*	Omnivorous	1	0.46		0.46		
*Metynnis sp.*	Omnivorous	3	0.17	0.11	0.18	0.05	0.28
*Myloplus sp.*	Herbivorous	1	0.03		0.03		
*Mylossoma sp.*	Herbivorous	70	0.07	0.04	0.06	0.02	0.19
*Nemadoras sp.*	Omnivorous	48	0.44	0.23	0.43	0.07	0.90
*Opsodoras sp.*	Omnivorous	8	0.38	0.20	0.29	0.20	0.78
*Oxydoras sp.*	Omnivorous	10	0.13	0.09	0.10	0.06	0.30
*Parauchenipterus sp.*	Insectivorous	5	0.10	0.07	0.10	0.04	0.22
*Pellona sp.*	Carnivorous	23	0.72	0.39	0.66	0.19	1.56
*Piaractus sp.*	Herbivorous	1	0.11		0.11		
*Pimelodus sp.*	Omnivorous	108	0.21	0.09	0.20	0.05	0.53
*Pinirampus sp.*	Carnivorous	10	1.55	0.40	1.64	0.79	2.03
*Plagioscion sp.*	Carnivorous	11	0.59	0.19	0.63	0.34	0.83
*Potamorhina sp.*	Detritivorous	149	0.12	0.05	0.11	0.01	0.41
*Prochilodus sp.*	Detritivorous	53	0.09	0.06	0.07	0.02	0.41
*Psectrogaster sp.*	Detritivorous	69	0.15	0.11	0.13	0.03	0.89
*Pseudoplatystoma sp.*	Carnivorous	7	0.63	0.52	0.41	0.25	1.74
*Pterodoras sp.*	Herbivorous	5	0.01	0.00	0.01	0.01	0.01
*Pterygoplichthys sp.*	Detritivorous	13	0.08	0.04	0.07	0.03	0.16
*Pygocentrus sp.*	Carnivorous	30	0.26	0.15	0.21	0.07	0.81
*Rhaphiodon sp.*	Carnivorous	39	1.19	0.97	1.03	0.21	6.06
*Rhytiodus sp.*	Herbivorous	3	0.02	0.01	0.02	0.02	0.03
*Roeboides sp.*	Carnivorous	3	0.33	0.20	0.38	0.11	0.49
*Roestes sp.*	Carnivorous	3	0.42	0.18	0.40	0.25	0.61
*Satanoperca sp.*	Insectivorous	8	0.13	0.04	0.14	0.06	0.19
*Schizodon sp.*	Herbivorous	18	0.27	0.25	0.13	0.01	0.76
*Semaprochilodus sp.*	Detritivorous	5	0.16	0.05	0.15	0.10	0.23
*Serrasalmus sp.*	Carnivorous	59	0.41	0.31	0.34	0.02	1.70
*Sorubim sp.*	Carnivorous	19	0.57	0.21	0.57	0.22	0.97
*Tatia sp.*	Carnivorous	1	0.08		0.08		
*Trachydoras sp.*	Omnivorous	1	0.18		0.18		
*Triportheus sp.*	Omnivorous	99	0.28	0.25	0.17	0.04	1.04
*Zungaru sp.*	Carnivorous	2	0.75	0.46	0.75	0.43	1.08

### 3.3. Discussion

This study represents one of the largest surveys of riverine populations in the Brazilian Amazon with respect to hair-Hg concentrations. Despite the changes of economic development that is occurring in Western Amazon, fish is still the main source of protein and the principal means of exposure to MeHg for riverines of the Madeira river. Elevated hair-Hg concentrations, according to WHO guidelines (2008) were found in all studied communities. Fish consumption habits influence HHg and is influenced by lifestyle (isolation and subsistence on forest and river resources). Nevertheless, this survey suggests that overall fish consumption (HHg) has diminished in comparison to studies done in the last 15 years [19,20,21]. In the Brazilian Amazon few studies on Hg exposure in riverine populations have addressed the issue of health effects based on epidemiological study (longitudinal cohort study on early childhood Hg exposure. This is due to the difficulty in working in remote regions (long distance from the urban areas, where the transportation usually is by boat). Yet, in these remote areas the population's access to health services is almost non-existent, the deliveries are made by midwives and Hg exposure in children, women of child-bearing and pregnant women have been a cause of great concern because these groups are particularly vulnerable. We know that during the pregnancy MeHg readily crosses the placental barrier and also because the toxicological effects of Hg exposure are more serious in the developing central nervous system of children than in adults [22]. 

Comparative large studies of general populations of the Amazon have previously showed higher median HHg values [3]. In our study women between the ages of 16 and 49 years (childbearing females) have median HHg ranging from 2.4 to 5.3 ppm, depending on subsistence living conditions. Comparing these results with an exposure of 1µg/g in hair for female of childbearing age in the Borum *et al*. [23], our results are substantially high in the remote area of Resex Cuniã. However, no peak exposures from freshwater fish species with high Hg concentrations were observed in this study.

In the present study, few hair samples (1.2%) reached values below 1 ppm in the Cuniã subsistence community, while the other areas showed circa 13% (Areas 2 and 3), or 18 to 20% (respectively for Areas 4 and 1). From a practical standpoint, Hg exposure (HHg) in Amazonian women and children is exclusively from eating fish; other forms of organic mercury (ethylmercury) do exist for young children that are immunized with thimerosal-containing vaccines [24]. However, it is worth noting that the small amounts of ethylmercury in vaccines are not sufficient to be differentiated in hair of children that have been vaccinated for more than three months [25].

Also, for susceptible subgroups (women of pregnant age and young children) Andean Amazon women showed a range of median values (3.3 to 5.5 ppm) that were comparable to ours [5]. However, in French Guyana, maternal HHg ranged from 2.8 to 12.7 [26]. For women in different parts of the Brazilian Amazon, almost all studies conducted in the Basins of the Negro river [27,28] and the in Tapajós River Basin [29,30,31,32,33,34,35,36,37,38] showed higher HHg concentrations than in our current study; an exception was found in women of Alta Floresta [39]. Previous studies in the Madeira river have also shown higher median values than the present study [27,40,41,42]. 

There is general agreement that environmental MeHg exposure during pregnancy poses a significant risk to children’s neurodevelopment [43]. The threshold of maternal HHg concentration that may cause adverse effects to the fetus is in the order of 10–20 ppm [44]. However, the potential sub-clinical health effects associated with this variation of exposure in riverine Amazon population are unclear.

The fish species consumed by subsistence Amazonian riverines depend on the catch, which varies with location and season. Although predatory species carry the highest concentrations of Hg, the predominantly available taxa in the riverines’ daily diet have much lower Hg concentrations [10]. In the present study, out of the 106 taxa sampled from the Madeira river, the median Hg concentrations in 17.7% exceeded the 0.5 µg/g WHO/FAO limit [45].

Innumerable studies have taken a simplistic approach, classifying fish as piscivorous/carnivorous to represent species at the top of the food chain. This is usually done in studies with a restricted number of taxa or reduced number of samples [9]. In the current study fish were classified more rigorously and the large sampling (N = 1,615) allowed an appraisal of fish availability for the studied areas. Additionally, this rigorous classification as a function of fish feeding behavior revealed patterns of variability in Hg concentrations. High Hg levels in piscivorous and carnivorous species are also accompanied by great variability, and together constitute only 30% of the catch (Figure 4), whereas fish with much lower median Hg concentrations (omnivorous and detritivorous) make up 53% of the catch. Therefore, depending on the frequency of consumption (which is a function of availability for subsistence riverines), risk analysis should take such facts in consideration.

Nutrition education and changes in type of fish have been tried for riverines of the Eastern Amazon [46]. However, changes in lifestyle may not translate into the perception of a better quality of life [47] and may be disruptive for their strategies of food security, with a possible compromise of nutritional status. Our ongoing studies with this population aim to analyze collected data addressing different biomarkers of nutritional status and health problems associated with a fish-based diet, principally MeHg exposure and specific outcomes, *i.e.*, pinpointing which benefits and health risks can be evaluated [48]. These studies will clarify what is needed if dietary interventions are to be recommended or implemented.

One of the strength of this study is the large sample size of the population and the high acceptance of the invited communities to participate in this study (>80%). This representation was crucial to assess Hg concentrations in human hair and in fish consumed by riverine communities before the impoundment of reservoir. Our findings are particularly important because they show the current Hg exposure scenario and how some communities, special the ones more isolated from the urban center, depend on the fish as a source of protein. Therefore, some changes in lifestyle (especially in the riverine communities to be resettled) most probably will reflect in differences in mercury exposure (HHg) between groups. However, personal health consequences are still being evaluated. The weakness is that we cannot yet interpret the health impact of the changes in lifestyle, regarding the benefits of fish in the traditional diet. 

## 4. Conclusions

This study does not show up to now any impact of the hydroelectric power plant. It was carried out before the reservoir flooding. Currently available fish in the Madeira river show a wide variability in Hg concentrations within historical reported values, also regarding the trophic level. Despite cultural similarities, riparians showed HHg distribution patterns that reflect changes in fish-eating habits driven by subsistence living brought on by socioeconomic changes in the region. However, a marked change in fish-eating habits probably will be verified in the resettled communities closest to the urban area of Porto Velho in few years. 
